# Supplementary material for: Association between mobility, non-pharmaceutical interventions, and COVID-19 transmission in Ghana: A modelling study using mobile phone data
Source: PLOS Glob Public Health. 2022 Sep 13;2(9):e0000502. doi: 10.1371/journal.pgph.0000502 (PMC10021296; doi:10.1371/journal.pgph.0000502)
Supplement: Attachment — Submitted filename: response_to_reviewers.docx [file pgph.0000502.s002.docx]

Journal Requirements:

1. Please provide separate figure files in .tif or .eps format only. Please ensure that all files are under our size limit of 20MB.

For more information about how to convert your figure files please see our guidelines: Once you've converted your files to .tif or .eps, please also make sure that your figures meet our format requirements

https://journals.plos.org/globalpublichealth/s/figures

| Response: | We have re-uploaded our figures as .tif files. |
| --- | --- |

2. Please amend your detailed Financial Disclosure statement. This is published with the article, therefore should be completed in full sentences and contain the exact wording you wish to be published.

iii). State what role the funders took in the study. If the funders had no role in your study, please state: “The funders had no role in study design, data collection and analysis, decision to publish, or preparation of the manuscript.”

| Response: | We have updated our Financial Disclosure Statement to include the above text. |
| --- | --- |

3. We have noticed that you have uploaded supporting information but you have not included a list of legends. Please add a full list of legends for all supporting information files (including figures, table and data files) after the references list.

| Response: | We have updated our manuscript to include a full list of legends for our supplemental information. |
| --- | --- |

4. Please provide us with a direct link to the base layer of the map used in Fig 3 and ensure this location is also included in the figure legend.

Please note that, because all PLOS articles are published under a CC BY license (creativecommons.org/licenses/by/4.0/), we cannot publish proprietary maps such as Google Maps, Mapquest or other copyrighted maps. If your map was obtained from a copyrighted source please amend the figure so that the base map used is from an openly available source.

Please note that only the following CC BY licences are compatible with PLOS licence: CC BY 4.0, CC BY 2.0 and CC BY 3.0, meanwhile such licences as CC BY-ND 3.0 and others are not compatible due to additional restrictions. If you are unsure whether you can use a map or not, please do reach out and we will be able to help you.

The following websites are good examples of where you can source open access or public domain maps:

* U.S. Geological Survey (USGS) - All maps are in the public domain. (http://www.usgs.gov)

* PlaniGlobe - All maps are published under a Creative Commons license so please cite “PlaniGlobe, http://www.planiglobe.com, CC BY 2.0” in the image credit after the caption. (http://www.planiglobe.com/?lang=enl)

* Natural Earth - All maps are public domain. (http://www.naturalearthdata.com/about/terms-of-use/)

| Response: | We have updated Figure 3 to use a basemap from the Humanitarian Data Exchange: https://data.humdata.org/. This map layer has a Creative Commons Attribution for Intergovernmental Organisations License (CC BY-IGO). The underlying map data is publicly available: https://data.humdata.org/dataset/cod-ab-gha. |
| --- | --- |

Please review your reference list to ensure that it is complete and correct. If you have cited papers that have been retracted, please include the rationale for doing so in the manuscript text, or remove these references and replace them with relevant current references. Any changes to the reference list should be mentioned in the rebuttal letter that accompanies your revised manuscript. If you need to cite a retracted article, indicate the article’s retracted status in the References list and also include a citation and full reference for the retraction notice.

| Response: | We have updated the reference list to cite the publication version of articles which were formerly preprints. |
| --- | --- |

Additional Editor Comments (if provided):

The authors present a very interesting analysis with novel data sources. There are comments that need to be addressed before it is suitable for publication. The last paragraph in the introduction is more suitable for the methods section, the objective needs to be more clearly specified, there needs to be some information on the SARS-CoV-2 testing strategy and accessibility during the study period in the methods, and clarity on how the model performance was evaluated.

| Response: | Thank you for your review of our work. We have expanded the description of the objectives of the study in the introduction:  *Here we estimate the relationship between the transmission of COVID-19 in Ghana and the changing NPIs introduced to mitigate the epidemic. We also estimate the effect of NPIs on transmission using human mobility data, which is a measurable proxy for responses to NPIs.*  We have also removed the final section of the introduction and incorporated portions of this paragraph in appropriate sections of the methods.  Additionally, we have included more information in the methods section about the varying availability of testing in Ghana during the early COVID-19 epidemic:  *COVID-19 testing was not equally available in all parts of Ghana during the COVID-19 epidemic and line list data includes many districts without complete time series in the early epidemic. Any testing arriving into a community after the introduction of COVID-19 may be biased by reporting both existing and newly acquired infections. To ensure the quality of data during the early stages of the COVID-19 outbreak in Ghana, we limited R_t_ estimation to 27 districts with reported cases before March 30th, 2020 (the beginning of the partial lockdown in Ashanti and Greater Accra), and at least 100 reported cases during the entire study period (12th March to 1st September 2020).*  We updated our method for quantifying the uncertainty of model parameters and have added the following on how model performance was evaluated:  *We quantified the uncertainty of model parameters using bootstrap resampling by creating 500 resampled datasets and retraining our model for each period and each resampled dataset. We then constructed 95% and 50% confidence intervals of model parameters by aggregating model parameter estimates for all resampled datasets trained for each period.* |
| --- | --- |

Reviewers' comments:

Reviewer's Responses to Questions

Comments to the Author

Reviewer #1: I suggest making clear the objective: In this article, the objective is to assess the combination of human mobility and NPI data to estimate their relationship over time with the progress of the COVID-19 97 epidemic in Ghana.

The analysis was carefully carried out, considering adjustments for holidays

Despite not including the period with Alpha or Beta variants, the manuscript had significant results for the start of the pandemic. Mobility and NPI stringency was able to explain variance in Rt during the early epidemic but this pattern declined as the epidemic progressed.

I suggest reviewing the formatting of table 1. It's like a picture.

| Response: | Thank you for the suggestion. We have updated the objective statement in the introduction to make the objective of this study more clear:  *Here we estimate the relationship between the transmission of COVID-19 in Ghana and the changing NPIs introduced to mitigate the epidemic. We also estimate the effect of NPIs on transmission using human mobility data, which is a measurable proxy for responses to NPIs.* |
| --- | --- |

Reviewer #2: This manuscript studies the relationship between human mobility, non-pharmaceutical interventions, and COVID transmission in Ghana. This work shows well-conducted research and the manuscript is well-written. I believe some points on the validation of the experimental results and the uncertainty characterization of the model should be clarified before it could be published.

First, it is unclear to me what kind of validation was used in the experiments to show the association between the case increases/decreases and the non-pharmaceutical interventions. Are these results temporal cross-validated using future datasets outside of the training set?

Second, how is the uncertainty in the proposed method evaluated and controlled in this paper? For example, Shea et al (2020) (Shea, Katriona, et al. "COVID-19 reopening strategies at the county level in the face of uncertainty: Multiple Models for Outbreak Decision Support." medRxiv (2020).) aggregated multiple models to capture the uncertainty of individual models in characterizing the association between the outbreak of COVID and interventions by policy makers.

| Response: | Thank you for your suggestions to improve this analysis. With respect to the uncertainty of the model parameters, we have updated our method to better present uncertainty around parameter estimates. In the updated manuscript, we include bootstrap resampling of districts to provide a qualitative assessment of parameter uncertainty.  We have expanded on this in the methods section:  *We quantified the uncertainty of model parameters using bootstrap resampling by creating 500 resampled datasets and retraining our model for each period and each resampled dataset. We then constructed 95% and 50% bootstrap confidence intervals of model parameters by aggregating model parameter estimates for all resampled datasets trained for each period.*  We have updated the results in Figure 4, Table 1 and in the supplemental Figures 13 and 17 and Tables 3 and 4 to include these estimates.  We feel that this presentation provides a more robust assessment of uncertainty compared to the previous approach which provided uncertainty around the results of a single model and we thank the reviewer for this suggestion. Our approach to quantifying uncertainty applies to one statistical model construction, whereas the approach presented in Shea et. al. (2020) – where different modeling groups contribute to an aggregate modeling result – provides a detailed understanding of scientific uncertainty around transmission dynamics. The Shea ensemble method is well suited to mechanistic models (which make up all but one of the models used in their paper) because the assumptions of these methods are more complex. Nonetheless, the ensemble method could be used to quantify uncertainty from multiple statistical models. In the spirit of this method, the bootstrap resampling generates multiple subsamples of districts and adds pooled uncertainty from the associations between *R_t_* and interventions, as suggested by the reviewer.  Our model does not include temporal cross validation as the aim of our analysis is an explanatory model of the association between *R_t_*, NPIs and Mobility during the early COVID-19 epidemic in Ghana. Because of varying patterns of movement behavior and changes in adherence to NPIs, we do not expect associations between *R_t_*, NPIs and mobility to remain constant during the study period. Rather, we are interested in presenting variations in these modeled associations during the early epidemic period and recognise that the results of our model are not applicable to prediction of *R_t_* outside of the study period. |
| --- | --- |

6. PLOS authors have the option to publish the peer review history of their article (what does this mean?). If published, this will include your full peer review and any attached files.

| Response: | Yes. We would like the peer review history of this article to be published. |
| --- | --- |

While revising your submission, please upload your figure files to the Preflight Analysis and Conversion Engine (PACE) digital diagnostic tool, https://pacev2.apexcovantage.com/. PACE helps ensure that figures meet PLOS requirements. To use PACE, you must first register as a user. Registration is free. Then, login and navigate to the UPLOAD tab, where you will find detailed instructions on how to use the tool. If you encounter any issues or have any questions when using PACE, please email PLOS at figures@plos.org. Please note that Supporting Information files do not need this step.
